# Laboratory experiments reveal intrinsic self-sustained oscillations in ocean relevant rotating fluid flows

**DOI:** 10.1038/s41598-022-05094-1

**Published:** 2022-01-26

**Authors:** Stefano Pierini, Paola de Ruggiero, Maria Eletta Negretti, Ilana Schiller-Weiss, Julia Weiffenbach, Samuel Viboud, Thomas Valran, Henk A. Dijkstra, Joël Sommeria

**Affiliations:** 1 Department of Science and Technology, Parthenope University of Naples, Naples, Italy; 2 CNRS, Grenoble INP, LEGI, Univ. Grenoble Alpes, Grenoble, France; 3 GEOMAR Helmholtz Centre for Ocean Research, Kiel, Germany; 4 Department of Physics, Utrecht University, Utrecht, The Netherlands

**Keywords:** Climate sciences, Ocean sciences, Physical oceanography, Fluid dynamics

## Abstract

Several ocean Western Boundary Currents (WBCs) encounter a lateral gap along their path. Examples are the Kuroshio Current penetrating into the South China Sea through the Luzon Strait and the Gulf of Mexico Loop Current leaping from the Yucatan peninsula to Florida as part of the Gulf Stream system. Here, we present results on WBC relevant flows, generated in the world’s largest rotating platform, where the Earth’s sphericity necessary to support WBCs is realized by an equivalent topographic effect. The fluid is put in motion by a pump system, which produces a current that is stationary far from the gap. When the jet reaches the gap entrance, time-dependent patterns with complex spatial structures appear, with the jet leaking, leaping or looping through the gap. The occurrence of these intrinsic self-sustained periodic or aperiodic oscillations depending on current intensity is well known in nonlinear dynamical systems theory and occurs in many real systems. It has been observed here for the first time in real rotating fluid flows and is thought to be highly relevant to explain low-frequency variability in ocean WBCs.

## Introduction

Western Boundary Currents (WBCs), such as, for example, the Kuroshio Current and the Gulf Stream belonging to the subtropical gyres of the North Pacific and Atlantic Oceans, respectively^1,2^, are very intense ocean currents flowing along the western boundary of ocean basins. They owe their existence to the sphericity of the Earth, and consequent dependence of the Coriolis parameter on latitude represented by the so-called planetary $\beta$-effect^1,3,4^. Along with their respective open ocean extensions, WBCs play an important role on climate because of their meridional heat transports and corresponding air-sea interactions and of their fundamental role in the global ocean circulation.

**Figure 1:**
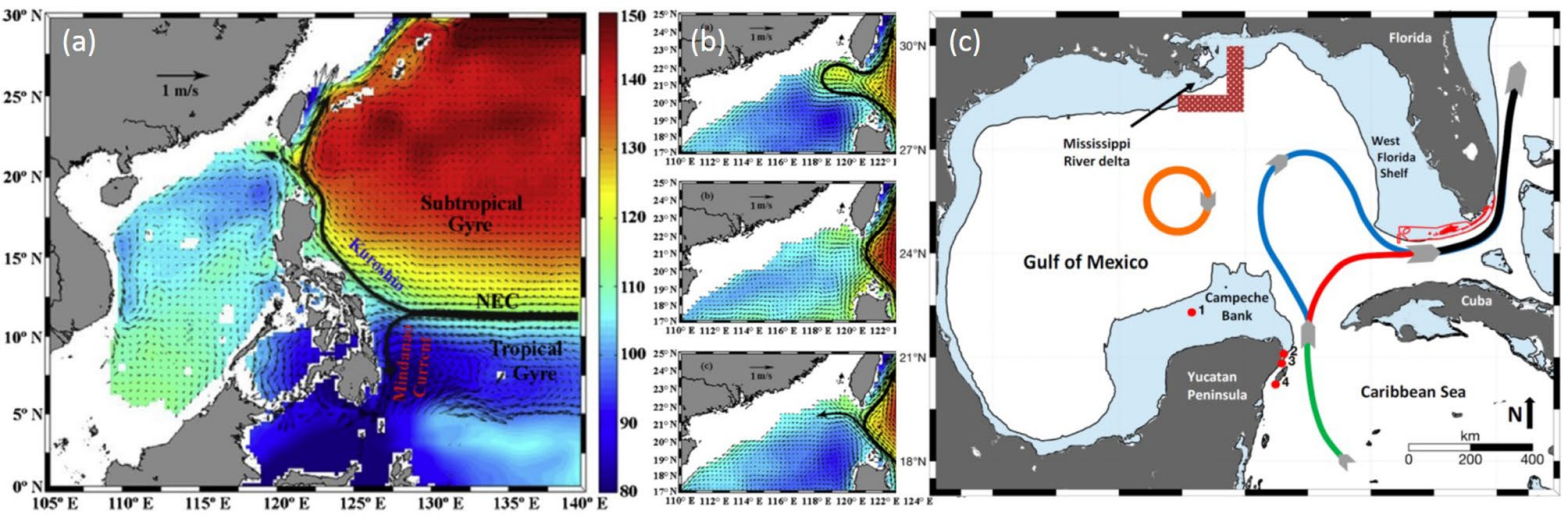
(**a**) Map of the Kuroshio intrusion into the South China Sea through the Luzon Strait (NEC stands for North Equatorial Current). (**b**) Sketch of the three types of Kuroshio intrusion, from top to bottom: looping, leaping and leaking path. (**c**) Sketch of the different states of the GoM Loop Current. Panels (**a**,**b**) are adapted from^5^, panel (**c**) is adapted from^6^.

One of the most interesting and intriguing WBC phenomena is the interaction of the current with a gap located along the western coast. Notable examples are the Kuroshio Current penetrating into the South China Sea (SCS) through the Luzon Strait^5^ (Fig. 1a,b) and the Gulf Stream leaping from the Yucatan peninsula to Florida, called the Gulf of Mexico Loop Current (GMLC)^6^ (Fig. 1c). A similar phenomenon occurs when the Kuroshio intrudes into the East China Sea (ECS) through the wider gap separating Taiwan to Japan^7^. A very important aspect is the different paths followed by the current when it penetrates west of the gap^5,6,8–10^: the Kuroshio can assume a looping, leaping or leaking path (Fig. 1b) while the GMLC can assume a retracted or an extended state (red and blue lines in Fig. 1c, respectively). A strong low-frequency variability accompanied by an energetic mesoscale eddy field is observed as well.

In order to assess the predictability of WBC variability in general—and in the specific case just discussed in particular—it is of fundamental importance to investigate whether the observed changes are mainly due to variations of the atmospheric forcing or, on the contrary, to mechanisms that are mainly internal to the ocean system. This is in fact a general issue of interest in many systems in Nature and Society, in which changes—said *intrinsic*, but also *internal*—are not the direct effect of a time-dependent external action but are rather due to mechanisms that are all internal to the system itself. Dynamical systems theory helps one formalize this phenomenon, that is common to virtually all fields of natural and social sciences (e.g.,^11–15^).

A dynamical system consists of a state space (with a finite or an infinite number of dimensions) whose points completely characterize the system and in which an (often) nonlinear evolution law is prescribed: thus, a trajectory in such a space provides the system’s evolution starting from a given initial point. Under some conditions, extreme dependence on initial data may occur, in which case the dynamical system is said to be chaotic. In a dissipative dynamical system, the energy is not conserved and a continuous input of energy from an external forcing is needed to sustain an equilibrium state. Dynamical systems can be either autonomous or nonautonomous depending on whether the parameters, including those in the external forcing, are constant or time-dependent. In the present paper we will focus on autonomous dissipative dynamical systems (ADDSs), which encompass a wide range of natural phenomena.

In ADDSs the volume of a subset of state space reduces in time on the average, thus leading to a time-invariant set called an attractor^12,13,16^. For example, for increasing values of a control parameter $\gamma$ the attractor may pass from a fixed point (i.e., a steady state) to a limit cycle (a periodic oscillation) past a critical value $\gamma _H$ called Hopf bifurcation; a further increase of $\gamma$ can eventually lead to a chaotic attractor through a series of local and/or global bifurcations^11,12^. Thus, the limit cycle represents the simplest form of self-sustained intrinsic variability (SSIV), i.e., of an intrinsic variability that is not a transient feature of the evolution. Aperiodic SSIV, often in the form of relaxation oscillations, occurs if the attractor is chaotic.

Disentangling the oceanic SSIV from the atmospherically forced oceanic variability is not an easy task but is fundamental for the interpretation, detection and attribution of the global oceanic variability and for assessing its predictability. The typical approach to this problem is to perform numerical simulations in idealized configurations (e.g.^17^), in idealized configurations with essential elements of realism (e.g.^18–24^) or in much more realistic settings (e.g.^25,26^). More recently, ensembles of parallel climate realizations^27^ are carried out to identify the oceanic SSIV (e.g.^28–30^). However, it is often difficult or even impossible to separate the intrinsic and forced forms of oceanic variability, as they are not at all independent in a highly nonlinear and nonautonomous (i.e., with time-dependent forcing) dynamical framework^27,31–33^. Nonetheless, it is always very useful to analyze ADDSs, in which, therefore, a constant-in-time climatological atmospheric forcing is imposed (this is what was in fact typically done in the so-called double-gyre problem of the wind-driven ocean circulation). The obvious limitation of this approach is that the variability thus obtained—though unambiguously intrinsic—may not necessarily find a real oceanic counterpart, but it at least provides plausible information about the underlying phenomenology and may even allow for significant model-altimeter data comparison (e.g., as done in^20,24,34^ for the Kuroshio Extension).

The SSIV of WBCs fits perfectly with the ADDS theory and has, in fact, been extensively analyzed in such a context within the double-gyre problem. A wealth of numerical process studies with different degrees of complexity and realism have been developed for this purpose based on a hierarchy of mathematical models (see^17,35,36^ for detailed reviews).

A question now arises: is there any evidence coming from laboratory experiments of cases of SSIV of WBCs? A more general question can even be posed: is there any evidence from laboratory experiments of SSIV of rotating fluid flows? It is worth stressing that (geostrophic) turbulence, modeled in many laboratory experiments with and without rotation, are themselves forms of intrinsic high-frequency variability of fluid flows, but they are not included in our definition because here we refer to coherent, large-scale flows yielding self-sustained low-frequency variability. Moreover, Rossby waves and modes are also forms of intrinsic variability in rotating fluids and have been modeled in rotating tank experiments^37–40^, however, they are not self-sustained motions but transient features excited by some time-dependent external forcing. Said this, the authors are not aware of any laboratory experiment performed with rotating platforms providing examples of SSIV of flows as defined above.

In this paper we present a laboratory study of the SSIV of WBCs encountering a lateral gap along its path. This provides the first experimental evidence based on laboratory experiments of SSIV of WBCs, but, more in general, of rotating fluid flows. The study has been carried out with the large rotating platform of LEGI-CNRS (Grenoble) in the framework of the 19GAPWEBS project of the European Union’s Horizon 2020 HYDRALAB+ program. The current is forced by a constant-in-time pumping system, thus the problem is cast in the framework of ADDSs.

It is worth noting that, apart from the fascinating opportunity of investigating experimentally a scaled representation of the real geophysical phenomenon, fluid dynamical experiments have the advantage—unlike numerical simulations—of offering a fine spatial and temporal resolution. On the other hand, only a reduced number of parameters can be controlled in the laboratory, and within limited ranges; thus, exact dynamical similarity with the real large-scale flow cannot be achieved. This does not prevent idealized experimental process studies to contribute substantially to shed light on the dynamical processes under investigation. In the specific case considered here, the large dimension of the Coriolis-LEGI rotating platform (13-m diameter, the world’s largest rotating facility) has allowed us to investigate the inertial regimes that characterize ocean dynamics, with little influence of viscosity. This aspect is essential for the occurrence of SSIV, that would otherwise be damped by viscous effects.

## Data and methods

### Experimental setup: motivation

The oceanographically generic problem of the interaction of a WBC with a lateral gap located along the western boundary was investigated in several observational^5,7,9,41,42^ and numerical model studies^43–45^. Laboratory experiments were also performed in small rotating tanks with a homogeneous^46,47^ and a two-layer fluid^48^. The experiments of Sheremet and Kuehl^46^ were carried out with a 1-m-diameter rotating basin, in which a cylindrical bathymetry accounted for the $\beta$-effect and a pumping system sustained the westward intensified jet. The main result was that, by slowly varying the pumping rate *q*, the WBC could either leap or penetrate the gap depending on whether inertial effects dominated the $\beta$-effect or not^43^. But at the same time another very interesting result was found: for the same value of *q*, say $q_0$, the jet could either leap or penetrate the gap depending on whether $q_0$ was reached for slowly decreasing or increasing values of *q*. This indicates the presence of a hysteresis phenomenon. It is worth noting that no SSIV was reported, in other words, for fixed *q* the states were steady. In the language of dynamical systems theory this means that the system admitted multiple fixed points and no Hopf bifurcation was passed. The lack of SSIV was likely related to the strong dissipation—provided in this case by bottom friction—associated with the small dimension of the basin.

The great relevance of the problem and the valuable results obtained with a small rotating tank, calls for further laboratory experiments that could possibly reveal SSIV associated with gap-leaping or intruding WBCs in the context of ADDSs. This however requires larger scale simulations and, in turn, a larger rotating tank facility. This has motivated the 19GAPWEB-HYDRALAB+ project, which was carried out with the 13-m-diameter Coriolis-LEGI rotating platform. The experimental setup was based on a substantial extension of three previous laboratory experiments performed by Pierini et al.^40^ with the old Coriolis rotating tank of CNRS-Grenoble and by Pierini et al.^49,50^ with the 5-m diameter Coriolis rotating tank of SINTEF in Trondheim (Norway).

### Experimental setup: geometry and instrumentation

**Figure 2:**
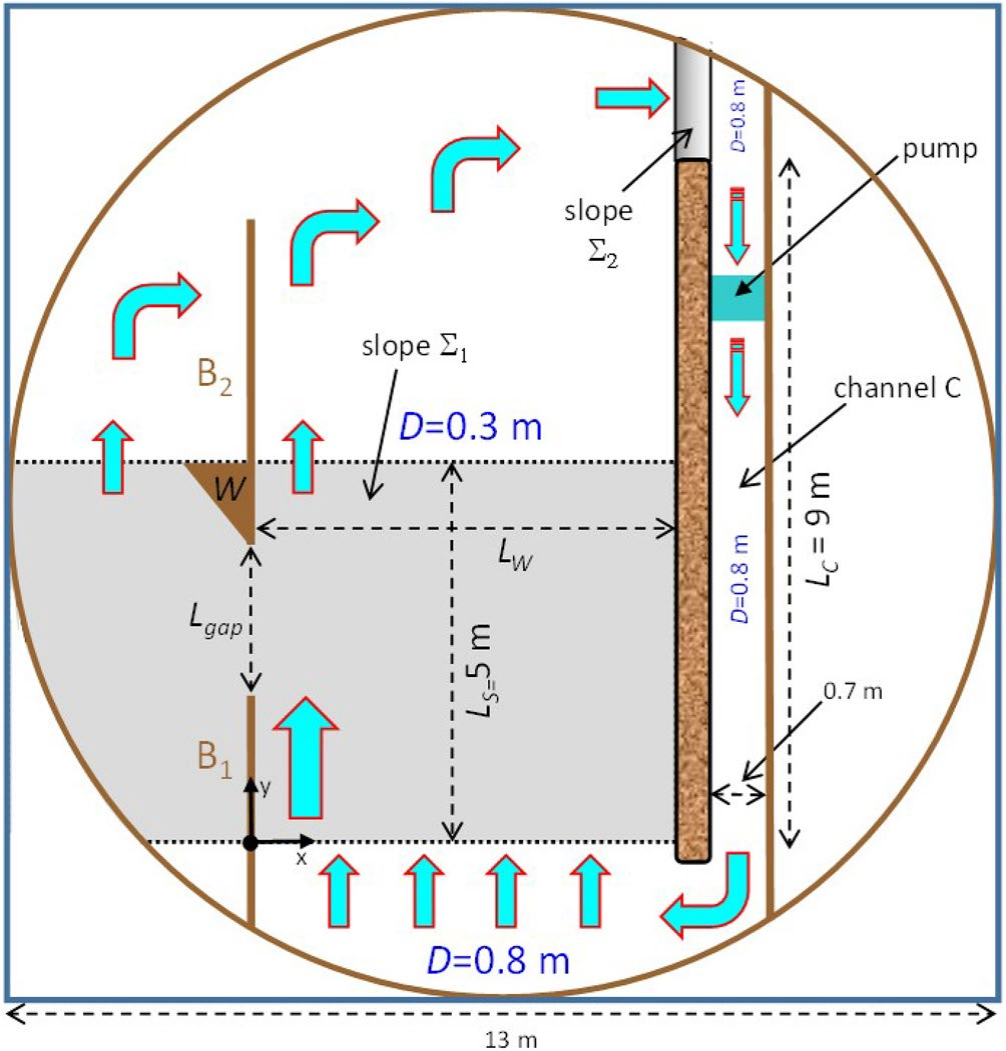
The laboratory setup (see the text for details).

The experimental setup is illustrated in Fig. 2 while a 3D sketch is reported in Supplementary Figure S1(a). A pumping system located in the channel *C* produces a current of constant speed *U* at its “southern” border (thus we are dealing with an ADDS); a virtually unsheared flow at the southern entrance of the topographic slope $\Sigma _1$ is thus formed. The bottom slope provides the topographic $\beta$-effect necessary for the intensification along the “western” boundary $B_1$. This can be understood as follows. By invoking the conservation of potential vorticity in the quasigeostrophic approximation^1^, one has

1 $$\begin{aligned} \zeta +f_{0}+\beta ^{*} y=const \end{aligned}$$

where $\zeta = v_{x}-u_{y}$ is the relative vorticity of the water column (subscripts denote differentiation), *u* and *v* are the current velocity components along *x* and *y*, respectively, $f_0=4\pi /T$ is the Coriolis parameter (*T* is the rotation period of the platform), $\beta ^{*} =f_0 {\gamma }'\left( y \right) / {\bar{D}}$ is the topographic $\beta$ simulating the corresponding planetary effect ($\gamma$ is the bottom topography, ’ indicates derivative and ${\bar{D}}$ is the mean slope depth) and *y* is the “northward” direction. By moving northward, a water column experiences an induction of negative (clockwise) relative vorticity from the planetary vorticity: at the entrance of the slope ($y=0$), we find $\zeta \simeq 0$, thus, at a given $y=y_0$ one has $\zeta \simeq -\beta ^{*}y_0$. Negative shear vorticity (i.e., the westward intensification) in a virtually rectilinear boundary current is therefore generated by the presence of the lateral boundary $B_1$. Moreover, positive shear vorticity is present in a thin frictional boundary layer along the wall due to the no-slip boundary condition. The flux of negative vorticity input continuously provided by the northward flow is balanced by dissipation associated with bottom and lateral friction, so that a constant WBC is present upstream (but sufficiently far from) the entrance of the gap (Fig. 2).

**Figure 3:**
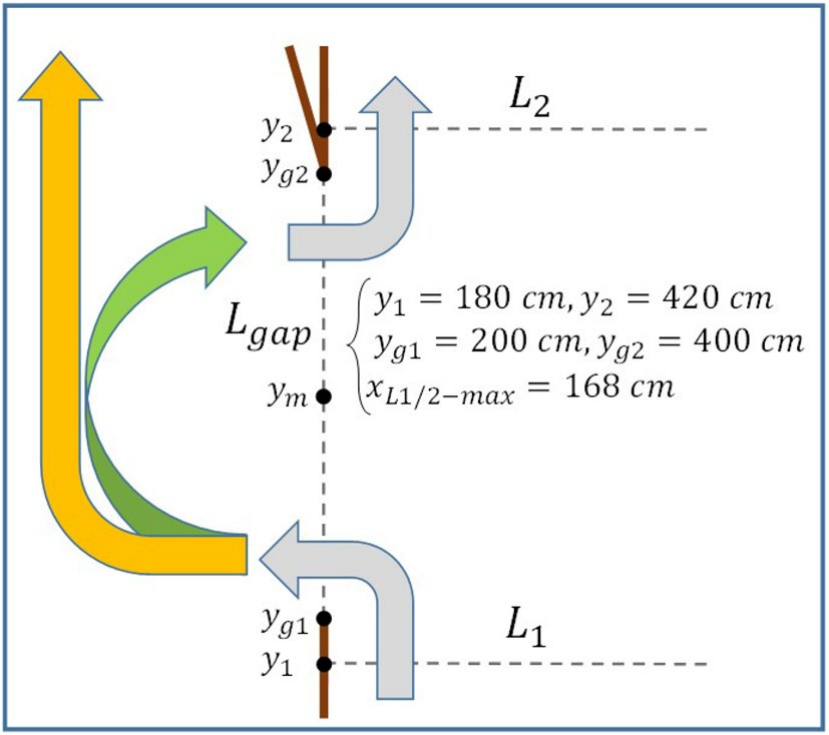
Definition of the three sections through which the fluxes are computed, along with additional information (see the text for details).

In the real ocean, the vorticity input is provided by an anticyclonic wind system, but the only way to mimic it in a rotating tank experiment is to apply a rotating rigid lid over the water surface^38^: this is not feasible with large rotating platforms, so a remote mechanical control was adopted in^49,50^, and the same is done here. Moreover, the use of a two-layer fluid would be necessary to account for baroclinic effects, but an inclined rigid lid would be required to impart the beta effect to the upper layer^48^: again, since a rigid lid cannot be introduced in our case, homogeneous water was used.

It must be stressed that, apart from the new fundamental feature constituted by the lateral gap, there is another very important difference with respect to the setup first introduced in^49^ and later applied in^50^: the flow inside the channel *C*, that was generated by a moving paddle in the previous experiments, is now produced by a pumping system. The great advantage lies in the possibility of performing experiments with almost unlimited duration, while the duration with the moving paddle was limited by the time the latter reached its southernmost limit. Pierini et al.^50^ showed that the spinup time required for highly nonlinear WBCs (i.e., for those that are more closely dynamically similar to real WBCs) is comparable to the maximum duration of the paddle movement. Using the pump has now allowed us to perform experiments that are much longer than the spinup time for even stronger WBCs. At the same time, very long experiments are also needed to reveal SSIV, whose detailed temporal evolution could not be observed with a moving paddle.

As far as the instrumentation is concerned, tracer particles embedded in the fluid were used for flow visualization in the horizontal plane. The use of a continuous 25W Spectra Physic laser and of an optical system with a $75^{\circ }$ Powell lens made it possible to obtain a horizontal laser slice at a height of 64 cm from the bottom. The images of the illuminated particles captured by four cameras were then analyzed through the Particle Imaging Velocimetry (PIV) to obtain two-dimensional instantaneous velocity fields.

In addition, an Acoustic Doppler Velocimetry (ADV) mounted at the southern end of channel *C* was used to record instantaneous velocity components at 2kHz at different points across the width of the channel during each experiment. ADV calibration allowed to establish the relationship between the pumping rate *Q*, with units as appearing in the pump power variator and ranging between 2 and 9, and the speed *U* at the channel exit. Supplementary Figure S2 shows that *U* (and, therefore, the WBC intensity) depends almost linearly on *Q*.

### Scaling and data analysis

To study the dynamic similarity between the laboratory experiments and the full-scale ocean phenomena one can rely on the evolution equation of potential vorticity in the quasigeostrophic approximation^1^ (valid for our experiments because the Rossby number for a typical simulated WBC is much less than unity) in its steady and dimensionless form^49^. In such equation three dimensionless parameters, $\varepsilon$, *E* and *F*, appear measuring the importance of nonlinear inertial effects and that of lateral and bottom friction, respectively^49,50^. These parameters can then be expressed in terms of three length scales: the inertial^51^ ($\delta _I$), viscous Munk^4^ ($\delta _M$) and viscous Stommel^3^ ($\delta _S$) boundary layer widths. Their definitions are reported in part A of the Supplementary Information.

The experiments carried out in the present laboratory study lie in the same inertial regime obtained in^49,50^, but even stronger nonlinear effects are attained over a long duration thanks to the new pumping system (see the discussion in the preceding subsection). This has allowed us to explore a wider range of nonlinear WBC intensity, so that the transitions to time-dependent flows could be investigated.

As far as the data analysis is concerned, two-dimensional maps of the current velocity field ${\mathbf {u}}=\left( u,v \right)$ provided by the PIV, time series of fluxes and Hovmöller diagrams of the relative vorticity $\zeta$ derived from ${\mathbf {u}}$ and of *u* are presented. The volume fluxes $F_1, F_2$ and $F_{gap}$ across the lines $L_1, L_2$ and $L_{gap}$ shown in Fig. 3 are computed as follows:

2 $$\begin{aligned}&F_{1/2}\left( t \right) =\int _{0}^{x_{max}}D\left( y_{1/2}\right) v\left( x,y_{1/2},t \right) dx, \end{aligned}$$

3 $$\begin{aligned}&F_{gap}\left( t \right) =-\int _{y_{g1}}^{y_{g2}}D\left( y\right) u\left( 0,y,t \right) dy. \end{aligned}$$

where *D*(*y*) is the local water depth.

For an almost symmetric flow through the gap, such as the one schematically depicted by the green arrow in Fig. 3, $F_{gap}\sim 0$, so an additional parameter is needed to measure the strength of WBC intrusion. In this case, the moment *M* of the flow across the gap is introduced:

4 $$\begin{aligned} M\left( t \right) =\frac{2}{y_{g2}-y_{g1}}\int _{y_{g1}}^{y_{g2}}D\left( y\right) u\left( 0,y,t \right) \left( y-y_{m} \right) dy. \end{aligned}$$

For a looping current that enters the gap in the south and exits to the north (green arrow of Fig. 3), a positive moment across the gap is expected. An oscillating moment implies that the current across the gap oscillates in strength or that its meridional structure varies.

## Results and discussion

**Figure 4:**
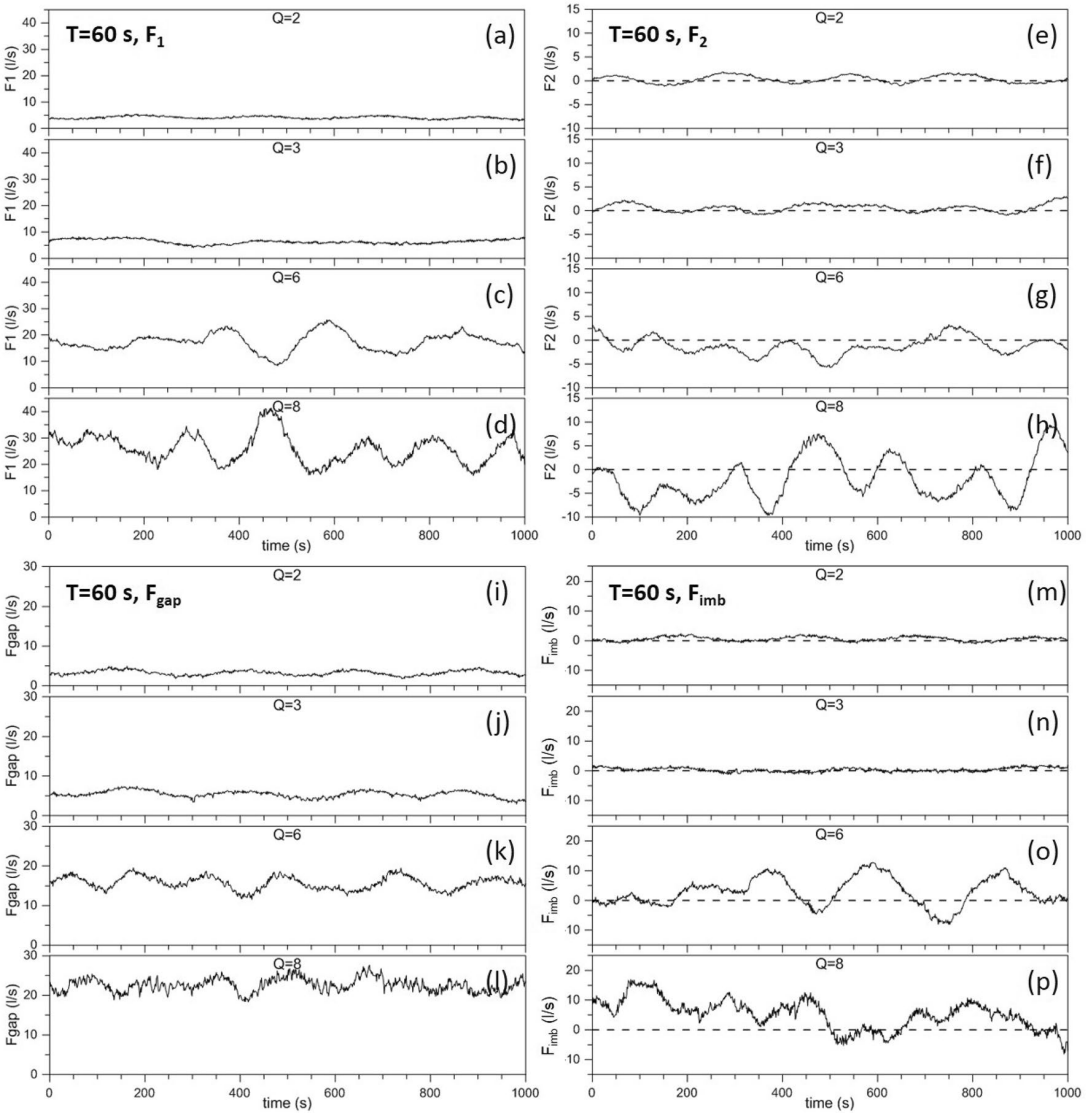
Time series of $F_1$ (**a**–**d**), $F_2$ (**e**–**h**), $F_{gap}$ (**i**–**l**), $F_{imb}$ (**m**–**p**) (units in $litres~s^{-1}$) for different values of *Q* (2, 3, 6, 8) and for a rotation period $T=60~\mathrm{s}$.

Experiments have been carried out by varying (i) the rotation period *T*, (ii) the pumping rate *Q* (and, consequently, the flow speed *U* at the channel exit and the WBC intensity) and (iii) the gap width $L_{gap}$. Here we shall present only results of experiments performed with a 2 m-wide gap (the results obtained with different gaps widths will be described elsewhere). We will consider four experiments that provide evidence of SSIV and a transition from almost periodic oscillations to aperiodic oscillations. They correspond to different values of *Q* but are all performed with a rotation period $T=60~\mathrm{s}$ (the corresponding results obtained with $T=30~\mathrm{s}$ are presented in the Supplementary Information). Figure 4 shows the time series of $F_1$, $F_2$, $F_{gap}$ and the volume flux imbalance $F_{imb}=F_1-F_2-F_{gap}$ during a 1000 *s* interval for different values of *Q* (2, 3, 6, 8).

Note that, based on the geometry of channel *C* and of the calibration curve reported in the Supplementary Figure S2, the values $Q=2,\;3,6,\;8$ correspond to the fluxes at the channel exit $F_C=4.32,\;7.78,\;20.3,\;30.3\;l/s$, respectively (*l* stands for *litre*). The flux $F_C$ is the most significant parameter to quantitatively characterize each experiment. However, in view of the qualitative nature of the present analysis and of the almost linear relation between *U* and *Q*, we will refer to the latter to provide a simple yet significant characterization of each flow.

The first thing to notice is that the water crossing the section $L_1$ recirculates, on average, mainly through the gap, while only a small fraction crosses $L_2$. For example, for $Q=2$, $\left\langle F_{1} \right\rangle =4.16~l/s$, $\left\langle F_{gap} \right\rangle =3.24~l/s$, $\left\langle F_{2} \right\rangle =0.31~l/s$ and, for $Q=8$, $\left\langle F_{1} \right\rangle =25.90~l/s$, $\left\langle F_{gap} \right\rangle =22.58~l/s$, $\left\langle F_{2} \right\rangle =-1.93~l/s$. In other words, on average, the water carried by the WBC reaches the shallow region (where $D=0.3~m$) mainly flowing west of boundary $B_2$ (Fig. 2, see also the orange arrow in Fig. 3) rather than through $L_2$ (but large fluctuations are present in $F_2$ as well). This flow behavior is compatible with the Kuroshio intrusion into the South China Sea through the Luzon Strait (Fig. 1a). To better represent the conditions in the Gulf of Mexico (Fig. 1c) a zonal boundary north-west of the gap inhibiting the recirculation west of $B_2$ could in principle be introduced: this would in turn force $\left\langle F_{gap} \right\rangle \approx 0$ and, therefore, $\left\langle F_{1} \right\rangle \approx \left\langle F_{2} \right\rangle$. However, an anomalous accumulation of water in such a semi-enclosed basin would result, with consequent emergence of spurious gravitational oscillations. Moreover, the manifestation of inertial effects and SSIV in the vicinity of the gap would also be artificially affected, but this should not be allowed as those are exactly the phenomena on which the present study is focused. Finally, it is worth noting that the mean flux imbalance is non-zero, e.g., $\left\langle F_{imb} \right\rangle =0.59~l/s$ for $Q=2$ and $\left\langle F_{imb} \right\rangle =5.26~l/s$ for $Q=8$: this is because part of the water flux crossing $L_1$ gives rise to a mesoscale eddy field over the slope east of the gap leading to an additional northward recirculation east of $L_2$.

The other fundamental aspects evidenced in Fig. 4 are the existence of SSIV and the transition from almost sinusoidal oscillations for small *Q* (and, therefore, for small current speeds, e.g., as shown in the Hovmöller diagrams below) to aperiodic oscillations for large *Q* (and, therefore, for larger current speeds), this behavior being consistent with the transition to chaos in a nonlinear dynamical system. For example, $F_{1}$ displays four small amplitude, almost sinusoidal cycles for $Q=2$ (Fig. 4a), but the oscillations become clearly aperiodic if the forcing amplitude is increased (e.g., see Fig. 4d for $Q=8$). Basically the same behavior is displayed for $F_2$, $F_{gap}$, $F_{imb}$.

It is also worth noting that, because the smallest pump intensity allowed by the system ($Q=2$) corresponds to a SSIV, steady state flows could not be simulated. In the language of dynamical systems this means that all our flows are beyond the Hopf bifurcation. Exactly the opposite occurred in the experiments performed in a small rotating platform by Sheremet and Kuehl^46^, in which the Hopf bifurcation could not be passed and all flows were steady. This stresses the fundamental role played by the large dimension of the Coriolis-LEGI rotating platform in producing flows that lie in the inertial regime typical of real ocean currents (see also the discussion in the introduction and in the section about the motivation).

**Figure 5:**
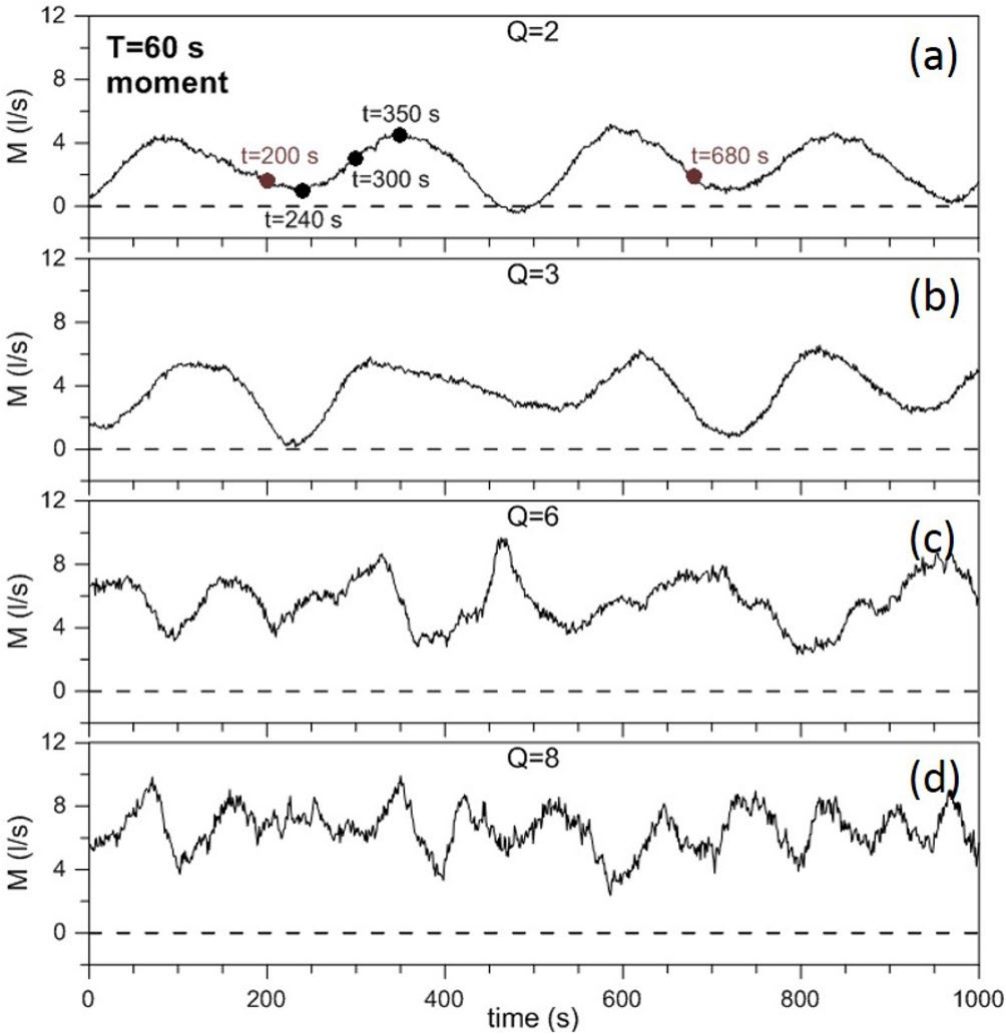
Time series of the moment *M* (in $litres~s^{-1}$) for different values of *Q* (2, 3, 6, 8) and for a rotation period $T=60~\mathrm{s}$.

When focusing on the gap section we have already noted that, in addition to $F_{gap}$, the moment *M* defined by Eq. (4) is also a significant parameter. Figure 5 shows the time series of *M* for the four values of *Q* and, again, for $T=60~\mathrm{s}$ (the corresponding results for $T=30~\mathrm{s}$ are shown in Supplementary Figure S4). The transition from periodic to aperiodic self-sustained oscillations is, again, striking. Note that, unlike for the fluxes (which give a direct information on the intensity of the fluctuations), the amplitude of the oscillations of *M* is almost independent of *Q*: this is due to the peculiar definition of this parameter.

**Figure 6:**
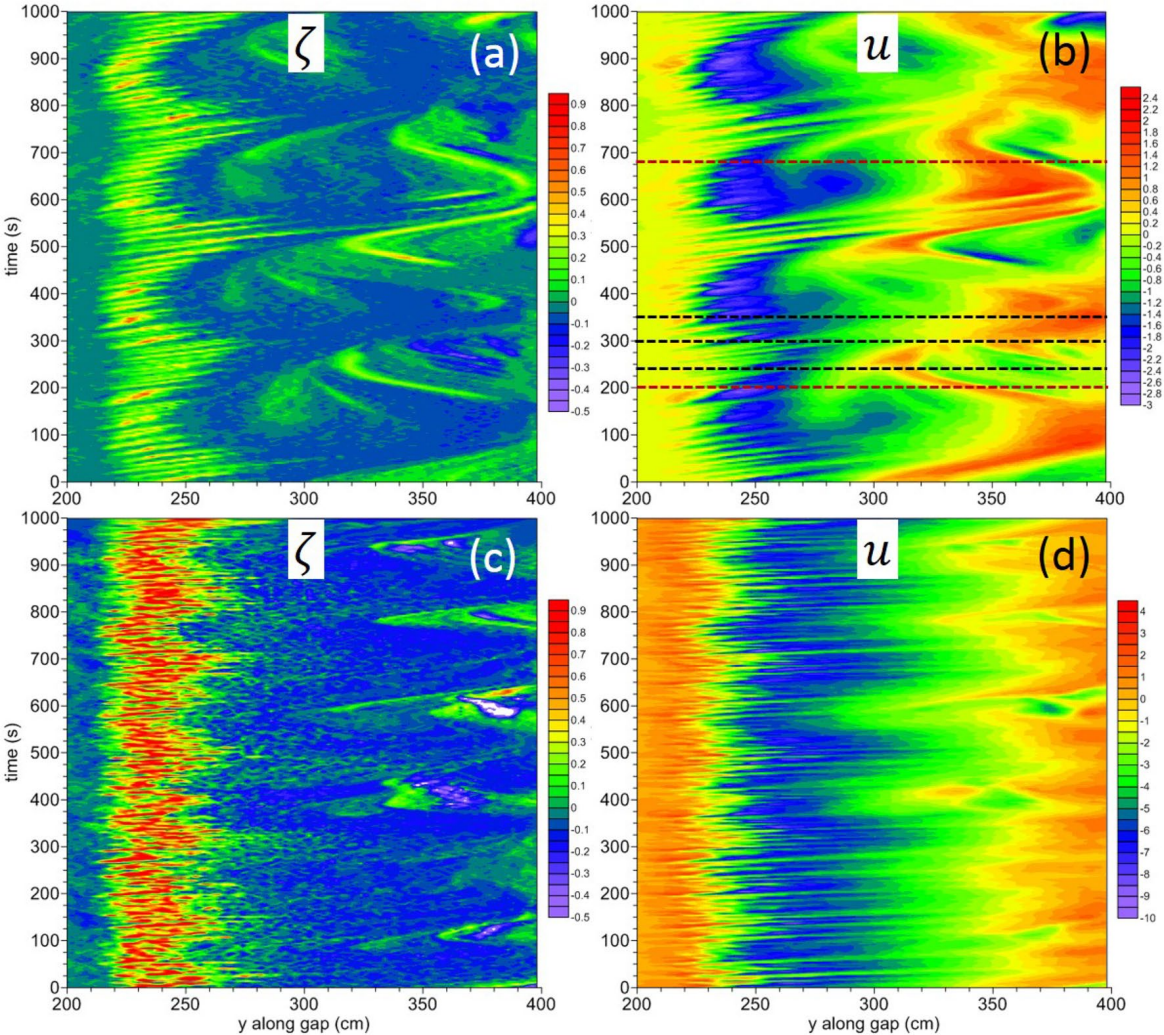
Hovmöller diagram of the relative vorticity $\zeta$ (in $s^{-1}$)—panel (**a**)—and current velocity *u* (in $cm~s^{-1}$)—panel (**b**)—for a rotation period $T=60~\mathrm{s}$ and $Q=2$. Panels (**c**,**d**): same but with $Q=8$. The velocity profiles of *u* along the black dashed lines in panel (**b**) are shown in the Supplementary Figure S6 and the corresponding snapshots of the 2D $\mathbf{u }$-field are shown in Fig. 7. The brown dashed lines in panel (**b**) correspond to the snapshots of Fig. 8b,c.

The Hovmöller diagrams of Fig. 6, showing the relative vorticity $\zeta$ and *u* along the gap for both $Q=2$ (panels a,b) and $Q=8$ (panels c,d), provide a more detailed picture of the SSIV. The variability in the nearly periodic case ($Q=2$) yields a predominant low-frequency component, with a period of about $250~\mathrm{s}$, e.g., as seen at $y=250~\mathrm{cm}$ in Fig. 6b, but a high-frequency component is present as well, with a predominant period of about $20~\mathrm{s}$, e.g., as shown by the striations in Fig. 6b; the inclination of the latter is evidence of northward propagation of the flow patterns, but features propagating in the opposite direction are also present, particularly in the range $y=300-400~cm$. In the aperiodic case ($Q=8$) the high-frequency component of the SSIV is stronger and is comparable in magnitude to the low-frequency component (e.g., compare Fig. 6a,b with Fig. 6c,d).

The analysis of the variability of the cross-gap current velocity *u* shown in Fig. 6b,d helps interpret the time series of Fig. 4. For example, at $y=250\;\mathrm{cm}$ (a location where the periodic vs. aperiodic character of the intrinsic fluctuations is more evident), for $Q=2$ *u* varies in the range $\sim (-1.8,\;0.2)\;\mathrm{cm/s}$ with an oscillation amplitude $\Delta \sim 2\;\mathrm{cm/s}$, while, for $Q=8$ one has $\sim (-7,\;-1)\;\mathrm{cm/s}$ and $\Delta \sim 6\;\mathrm{cm/s}$, respectively. It is worth noting that the ratio $\Delta _{Q=8}/\Delta _{Q=2}\sim 3$ is close to that observed for the fluxes $F_1$ and $F_2$ of Fig. 4 used to analyze the system’s SSIV. This confirms that the oscillation amplitude of those indicators is, in fact, representative of that of the local currents, evidencing the typical behavior of ADDS in which the transition from periodic to aperiodic (and possibly chaotic) SSIV is accompanied by an increase of its amplitude (if all the other parameters are unchanged).

In conclusion, Figs. 4, 5 and 6, provide striking evidence of SSIV in an ADDS in general—and in WBC dynamics in particular—in real rotating and ocean relevant fluid flows. The Supplementary Figures S3, S4, S5 correspond to Figs. 4, 5 and 6, respectively, for a rotation period $T=30~\mathrm{s}$ and show basically the same qualitative behavior. It is worth noting that in the nearly periodic case $Q=2$ the system shows smaller periods for both low- and high-frequency oscillations. Moreover, an intense intermediate-frequency oscillation can be detected in the temporal range $t=0{-}400~\mathrm{s}$ (cf. Supplementary Figure S5(b)).

**Figure 7:**
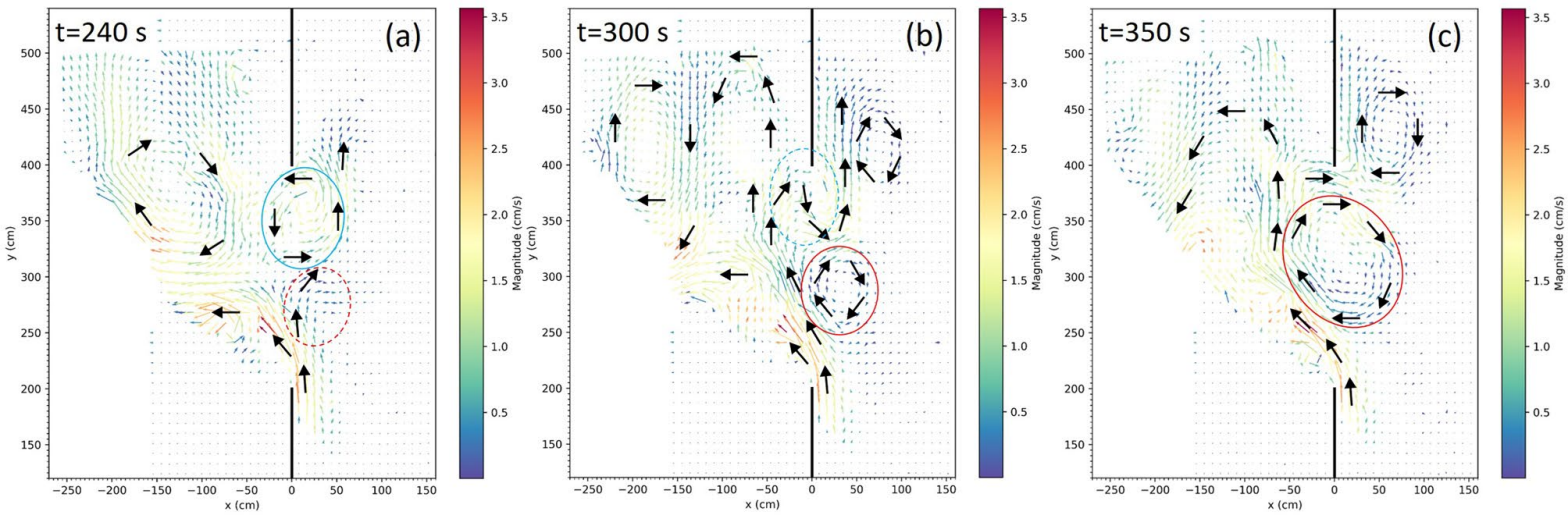
Snapshots of the current velocity field (in $cm~s^{-1}$) at $t=240~\mathrm{s}$ (**a**), $t=300~\mathrm{s}$ (**b**) and $t=350~\mathrm{s}$ (**c**) for $Q=2$ and a rotation period $T=60~\mathrm{s}$ (see the black dots in Fig. 5a, the $u-$field along black the dashed lines in Fig. 6b and Supplementary Figure S6 for the $u-$profiles along the gap).

We now analyze the spatial structure of the SSIV. The almost periodic case $Q=2$ is illustrated by the three snapshots of the current velocity field in Fig. 7, corresponding to the time instants indicated by black dots in Fig. 5a (big arrows and ovals are added to help visually capture the main flow features). The profiles of the “zonal” velocity *u* crossing the gap are reported in the Supplementary Figure S6 (the lines therein correspond to the $u-$field along the three black dashed lines in Fig. 6b). In addition, the almost periodic character of the main flow patterns of Fig. 7 is shown in the Supplementary Figure S7 and is discussed in part B of the Supplementary Information.

At $t=240~\mathrm{s}$ (Fig. 7a) the westward intensified northward flow intrudes through the gap at its southern border. This is due to the conversion of the positive shear vorticity present in the thin frictional boundary layer along the lateral wall in positive curvature vorticity when the wall suddenly disappears. The flow bifurcates at $y\simeq 250~cm$, producing a northward flow that bends eastward further north (at $y\simeq 280~cm$): this leads to the formation of an anticyclonic meander that retains the negative relative vorticity deriving from the westward intensification (cf. Fig. 6a). Besides, in the northern half of the gap a well defined cyclonic vortex is present: this gives little contribution to *M* and the same can be said for the southern half; this explains the small value of *M* in Fig. 5a and of $F_{gap}$ in Fig. 4i.

At $t=300~\mathrm{s}$ the meander has given rise to an anticyclonic vortex (red oval in Fig. 7b) while, conversely, the northern cyclonic vortex has given rise to a meander (cyan oval in Fig. 7b). Finally, at $t=350~\mathrm{s}$ (Fig. 7c) the anticyclonic vortex is now larger and stronger and is centered in the middle of the gap; moreover it is bounded by an outflow to the south and an inflow to the north. This flow structure is well represented by the green arrow in Fig. 3 and explains the large value of *M* in Fig. 5a.

**Figure 8:**
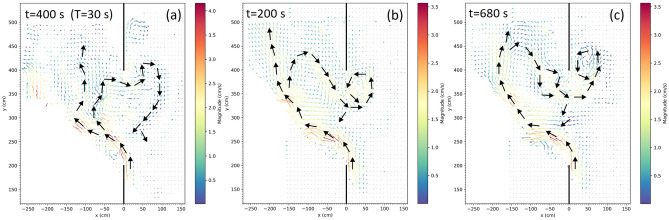
(**a**) Snapshot of the current velocity field (in $cm~s^{-1}$) at $t=400~\mathrm{s}$ for $Q=2$ and $T=30~\mathrm{s}$ (cf. Supplementary Figures S3–S5). (**b**,**c**) same but for $T=60~\mathrm{s}$ at $t=200~\mathrm{s}$ and at $t=680~\mathrm{s}$, respectively.

With reference to the oceanic phenomenon, the flows at $t=240,~300~\mathrm{s}$ do not fit into any of the three reference flows reported in Fig. 1b (looping, leaping and leaking path), but the one at $t=350~\mathrm{s}$ is an example of leaping path accompanied by leaking. A similar flow pattern can be identified in many experiments; Fig. 8a provides an example from an experiment with $T=30~\mathrm{s}$. Examples of looping paths are shown in Fig. 8b,c which, again, refer to $T=60~\mathrm{s}$ (see the brown dots in Fig. 5a and the brown lines in Fig. 6b). The two snapshots are separated by a $2T_p$ time lag, where $T_p\approx 240~\mathrm{s}$ is the approximate period of the oscillation. In both cases an elongated anticyclonic return flow intruding for a length comparable to that of the gap is evident, along with a northward leaking and a cyclonic vortex north-east of the gap. These paths are similar to those observed west of the Luzon Strait and in the Gulf of Mexico (cf. upper subpanel in Fig. 1b and blue line in Fig. 1c).

We have seen that such paths are transient features of a highly variable circulation, as happens in the oceanographic cases considered^8,10^. But it is worth stressing once more that the temporal variability obtained here is purely self-sustained, our system being autonomous, i.e., no changes in the external forcing and boundary conditions are imposed.

## Summary and conclusions

In this paper we have presented and discussed the results of laboratory experiments carried out with the world’s largest rotating platform aimed at simulating the behavior of a Western Boundary Current (WBC) encountering a lateral gap along its path. This is a relevant problem in physical oceanography which applies, among others, to two specific cases in the Northern Hemisphere: the Kuroshio Current penetrating into the South China Sea through the Luzon Strait and the Gulf of Mexico Loop Current. We have shown that the phenomena of leaking, leaping and looping of a WBC encountering a gap observed in the real ocean are well captured by our laboratory experiments.

The main focus of the present paper has been on the manifestation of very strong flow fluctuations—either periodic or aperiodic—which occur despite the fact that the forcing producing the WBC (a pump system) and all the boundary conditions are time-independent. This is a well known phenomenon typical of autonomous dissipative dynamical systems (ADDS) in which, under certain conditions, the interplay between the continuous energy input from an external source and its dissipation activate nonlinear mechanisms that generate self-sustained intrinsic variability (SSIV). Many observations and numerical simulations in all fields of Science have provided clear evidence of this phenomenon.

In this context, the present study is relevant in that it provides evidence—for the first time in laboratory experiments of rotating fluids—not only of SSIV in an ADDS, but also of the transition from almost periodic oscillations to aperiodic oscillations as the forcing intensity increases. In conclusion, our study illustrates nicely how a real complex system can produce coherent time-dependent changes merely by virtue of intrinsic mechanisms all internal to the system itself. It also motivates to further investigate the oceanic intrinsic low-frequency variability and to disentangle it—when possible—from the variability that is directly forced by the atmosphere^17,26,33^.

## Supplementary Information

Supplementary Information.
