# Supplementary material for: Laboratory experiments reveal intrinsic self-sustained oscillations in ocean relevant rotating fluid flows
Source: Sci Rep. 2022 Jan 26;12:1375. doi: 10.1038/s41598-022-05094-1 (PMC8792033; doi:10.1038/s41598-022-05094-1)
Supplement: Supplementary file 1 — Supplementary Information. [file 41598_2022_5094_MOESM1_ESM.pdf]

SUPPLEMENTARY INFORMATION FOR: Laboratory experiments reveal intrinsic self-sustained oscillations in ocean relevant rotating fluid flows

Stefano Pierini[1,*], Paola de Ruggiero[1], Maria Eletta Negretti[2], Ilana Schiller-Weiss[3], Julia Weiffenbach[4], Samuel Viboud[2], Thomas Valran[2], Henk A. Dijkstra[4], and Joël Sommeria[2]

[1]Department of Science and Technology, Parthenope University of Naples, Napoli, Italy
[2]Univ. Grenoble Alpes, CNRS, Grenoble INP, LEGI, Grenoble, France
[3]GEOMAR Helmholtz Centre for Ocean Research, Kiel, Germany
[4]Department of Physics, Utrecht University, Utrecht, The Netherlands
[*]stefano.pierini@uniparthenope.it

ABSTRACT

Several ocean Western Boundary Currents (WBCs) encounter a lateral gap along their path. Examples are the Kuroshio Current penetrating into the South China Sea through the Luzon Strait and the Gulf of Mexico Loop Current leaping from the Yucatan peninsula to Florida as part of the Gulf Stream system. Here, we present results on WBC relevant flows, generated in the world's largest rotating platform, where the Earth's sphericity necessary to support WBCs is realized by an equivalent topographic effect. The fluid is put in motion by a pump system, which produces a current that is stationary far from the gap. When the jet reaches the gap entrance, time-dependent patterns with complex spatial structures appear, with the jet leaking, leaping or looping through the gap. The occurrence of these intrinsic self-sustained periodic or aperiodic oscillations depending on current intensity is well known in nonlinear dynamical systems theory and occurs in many real systems. It has been observed here for the first time in real rotating fluid flows and is thought to be highly relevant to explain low-frequency variability in ocean WBCs.

Supplementary Figures

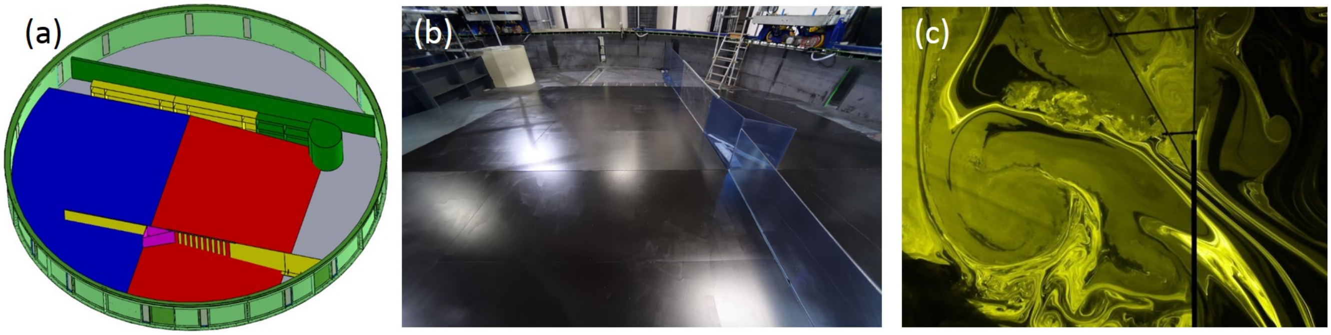

Supplementary Figure S 1. (a): A 3D perspective view of the experimental facility. (b): Photo showing an overall view of the experimental setup from downstream, with the western boundary to the right. (c): Example of flow visualized with Rhodamine fluorescent dye by the horizontal laser slice.

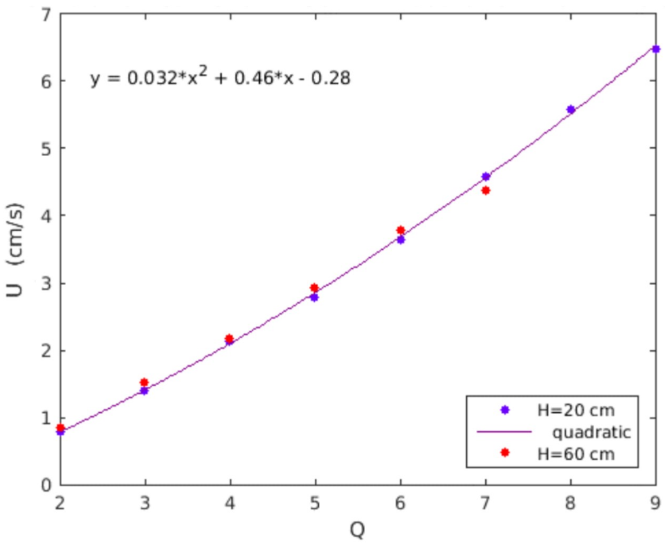

Supplementary Figure S 2. ADV probe calibration for $T = 30\ s$. Q is the pumping rate (with units as appearing in the pump power variator) and U is the flow speed at the channel exit. Measurements were taken at two different depths: $D = 20\ cm$ and $D = 60\ cm$).

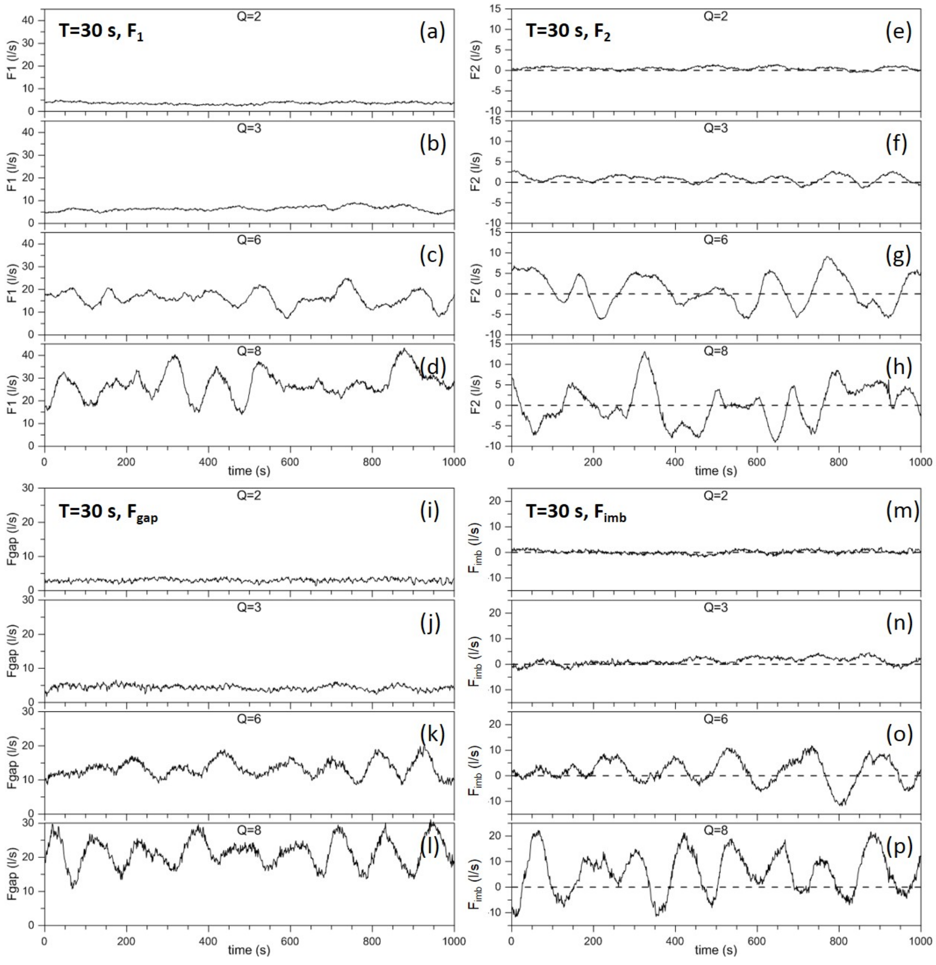

Supplementary Figure S 3. Time series of F_1 (a-d), F_2 (e-h), F_{gap} (i-l), F_{imb} (m-p) (units in $litres\ s^{-1}$) for different values of Q (2, 3, 6, 8) and for a rotation period $T = 30\ s$.

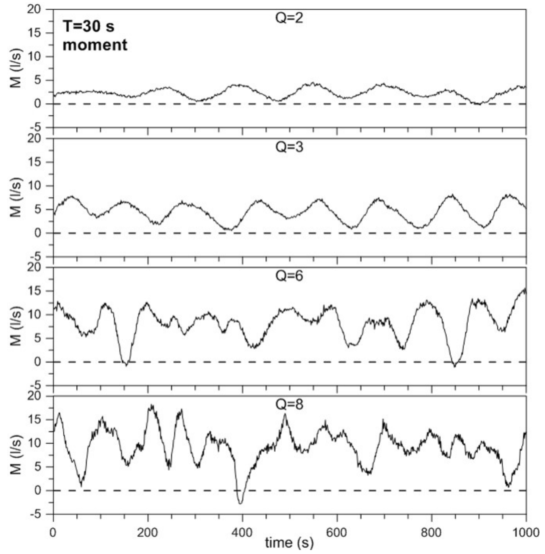

Supplementary Figure S 4. Time series of the moment M (in $litres\ s^{-1}$) for different values of Q (2, 3, 6, 8) and for a rotation period $T = 30\ s$.

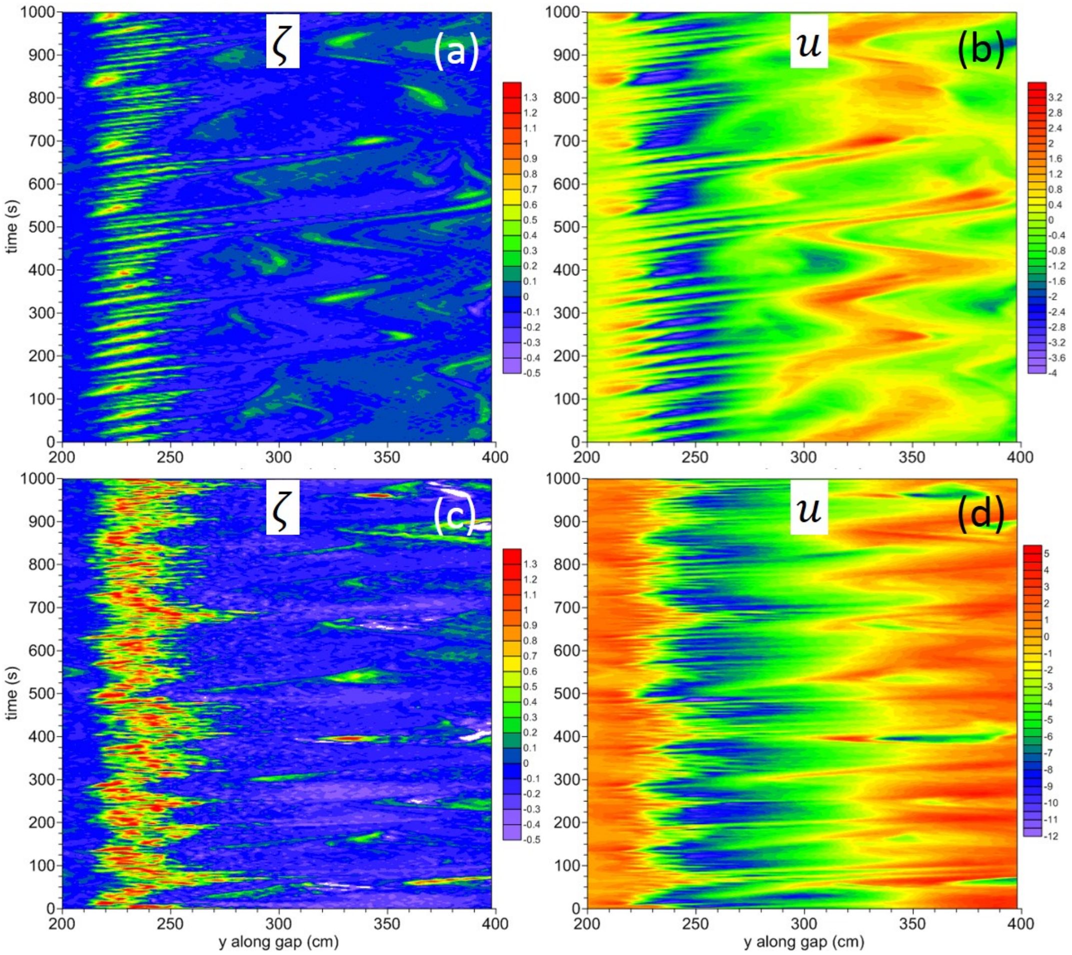

Supplementary Figure S 5. Hovmöller diagram of the relative vorticity ζ (in s^{-1}) –panel (a)– and current velocity u (in $cm\ s^{-1}$) –panel (b)– for a rotation period $T = 30\ s$ and $Q = 2$. Panels (c,d): same but with $Q = 8$.

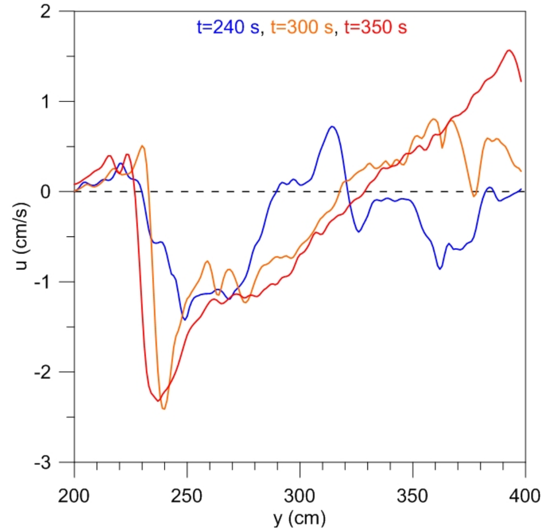

Supplementary Figure S 6. Profiles of u along the gap for a rotation period $T = 60\ s$ and $Q = 2$ at times $t = 240,\ 300,\ 350\ s$ (see the black dots in Fig. 4(a) and the $u-$field along the black dashed lines in Fig.5(b)).

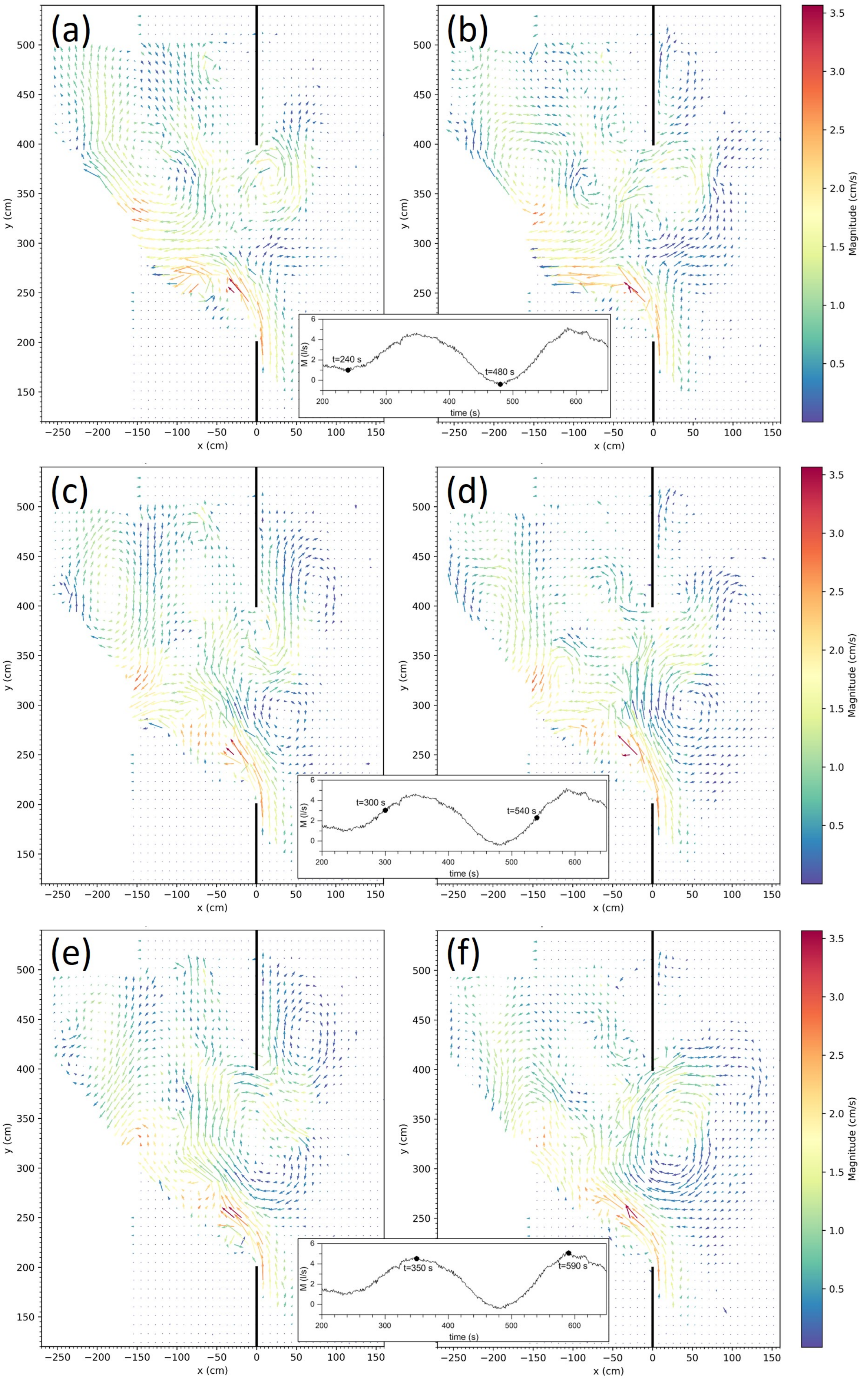

Supplementary Figure S 7. (a,c,e): Snapshots of the current velocity field (in $cm\ s^{-1}$) at $t = 240\ s$ (a), $t = 300\ s$ (c) and $t = 350\ s$ (e) for $Q = 2$ and a rotation period $T = 60\ s$. (b,d,f): same with a $240\ s$ time lag.

Supplementary information

A: "Scaling and data analysis"

Here we report the definition of the dimensionless parameters ε, E and F and of the boundary layer widths δ_I, δ_M and δ_S relevant for the study the dynamic similarity between the laboratory experiments and the full-scale ocean phenomena (for a detailed discussion see [49,50]). The parameters ε, E and F measure the importance of nonlinear inertial effects and that of lateral and bottom friction, respectively:

$$\varepsilon = \left(\frac{\delta_I}{l}\right)^2 ; E = \left(\frac{\delta_M}{l}\right)^3 ; F = \frac{\delta_S}{l}, \tag{S1}$$

where l is the WBC width. In (S1) the length scales

$$\delta_I = \left(\frac{U}{\beta}\right)^{1/2} ; \ \delta_M = \left(\frac{A_H}{\beta}\right)^{1/3} ; \ \delta_S = \frac{r}{\beta} \tag{S2}$$

provide the inertial [51] (δ_I), viscous Munk [4] (δ_M) and viscous Stommel [3] (δ_S) boundary layer widths, where r is the inverse of the spindown time due to bottom friction. For the real ocean, β is the planetary beta-effect, U is a typical zonal velocity scale and A_H is the lateral eddy viscosity coefficient. For our laboratory experiments, $\beta \to \beta^*$, U is the flow speed at the exit of channel C defined above and A_H is substituted by the kinematic molecular viscosity of water.

Thanks to the large dimension (5 m-diameter) of the rotating platform of SINTEF (Trondheim), Pierini et al. [49,50] were able to model highly nonlinear inertial WBCs, for which $l \sim \delta_I \gg \delta_M, \delta_S$. They also showed that these WBCs are basically dynamically similar to full-scale WBCs —such as the Gulf Stream and Kuroshio— in the inertial regime of width $l \sim \delta_I$ (where the predominant nonlinear terms balance the planetary vorticity gradient term), except in the thin viscous boundary layer of width $\delta_* = \sqrt{\delta_I A_H / U} \ll \delta_I$. The experiments carried out in the present laboratory study lie in the same inertial regime obtained in [49,50].

B: "Results and discussion"

We have already seen that the almost periodicity of the SSIV in the experiment with $Q = 2$ and $T = 60\ s$ deduced from the time series of the fluxes (Figs. 4(a,e,i,m)) and of the moment (Fig. 5(a)) is also found in the looping pattern appearing at $t = 200\ s$ and $t = 200\ s + 2T_p$ (Figs. 8(b,c)), where $T_p \approx 240\ s$ is the approximate period of the oscillation. Fig. S7 provides additional evidence of the near periodicity of the main flow patterns shown in Fig. 7 for $t = 240,\ 300,\ 350\ s$.

The left-hand column of Fig. S7 shows the same snapshots of the current velocity field of Fig. 7 while, in the right-hand column the corresponding snapshots for $t + T_p$ are shown for comparison (the location of each time instant in the moment time series is also reported for the sake of clarity). All the main patterns identified by the ovals in Fig. 7 are indeed present with little differences after one period. But also other secondary patterns correspond as well with small differences like, for example, the large anticyclonic vortex west of the gap in Figs. S7(a,b), the cyclonic-anticyclonic vortex pair north-west of the gap in Figs. S7(c,d), the cyclonic meander north-west of the gap in Figs. S7(e,f).

Differences are present as well. For example, some deformation of the main flow patterns, the different circulation east of boundary B_2 north of the gap and the presence/lack of small-scale features; the latter are likely generated by secondary instability mechanisms. But overall, a qualitative evaluation of flow structure confirms the substantial periodicity of the specific experiment.

In conclusion, by using the language of dynamical systems we note that the enormous number of degrees of freedom available in our real fluid flow collapse onto few, well organized and robust flow patterns exhibiting a self-sustained periodic oscillation. This provides a fascinating example of how nonlinear dynamical systems theory, whose mathematical methods were initially developed for systems with few degrees of freedom [11-15,35], allows interpreting the complex behavior of real physical phenomena.
